# Brain atrophy patterns in anti-IgLON5 disease

**DOI:** 10.1093/brain/awaf256

**Published:** 2025-07-12

**Authors:** Selina M Yogeshwar, Frederik Bartels, Thomas Grüter, Sergio Muñiz-Castrillo, Géraldine Picard, Yvette S Crijnen, Emilien Bernard, Anna Heidbreder, Anastasia Zekeridou, Marius Ringelstein, Andrea Kraft, Stjepana Kovac, Klaus-Peter Wandinger, Juna M de Vries, Agnita J W Boon, Sharon Veenbergen, Christian Geis, Loana Penner, Nico Melzer, Frank Leypoldt, Morten Blaabjerg, Sean J Pittock, Carles Gaig, Lidia Sabater, Joan Santamaria, Francesc Graus, Josep Dalmau, Harald Prüss, Romana Höftberger, Bettina Schreiner, Andrew McKeon, Jan Lewerenz, Sarosh Irani, Emmanuel Mignot, Maarten J Titulaer, Ilya Ayzenberg, Jérôme Honnorat, Carsten Finke, for the IgLON5 Imaging Consortium, Aurélie Méneret Isabelle Francillard, Dimitri Renard, Virginie Desestret, Nicolas Capet, Jeanbaptiste Davion, Julie Boucher, Helene Zephir, Philippe Damier, Helene Combres, Marie Rafiq, Fabrice Bonneville, Marina Cumin, Antoine Soulages, Mathilde Compoint, Maximilien Moulin, Edouard Berling, Eve Garrigues, Marine Chanson, Veronique Bourg, Caroline Giordana, Jeanne Benoit, Olivier Flabeau, Emmanuelle Derivoyre, Gilles Ryckewaert, Tifanie Alberto, Nicolas Carriere, Sohrab Mostoufizadehs, Melanie Barbay, Marie Benaiteau, Alberto Vogrig, Francois Sellal, Olivier Casez, Cedric Bruel, L Kalifa, Elena Camelia Rusu, Sarah Demortiere, Adil Maarouf, Adrien Wang, Adelaide Brasset, Clarisse Carra Dalliere, Arthur Attal, Céline Demourant, Florent Cluse, Jean-Luc Houeto, Norbert Brueggemann, Justina Dargvainiene, Thomas Seifert, Ulrich Hofstadt-van Oy, Michael Nagel, Christian Urbanek, Ina Schroeder, Peter Schramm, Silke Tonner, Caspar Seitz, Marlene Tschernatsch, Ha-Yeun Chung, Jonathan Wickel, Florian Schoeberl, Stefan Macher, Mateus M Simabukuro, Simone Zittel-Dirks, Brigitte Wildemann, Jan Kothaj, Carlos Ordas, Javier Villacieros Álvarez, Klemens Angstwurm, Stefan Macher, Evelyn Berger-Sieczkowski, Ángela Milán Tomás, Antonio Martin Bastida, Sonia Quintas, Nicola Tambasco, Pasquale Nigro, Raffaele Iorio, Yoya Ono, Takayoshi Shimohata, Kimura Akio, Takekoshi Akira, Mette Scheller-Nissen, Christian Hartmann, Danielle Bastiaansen, Robin van Steenhoven, Katia Schwichtenberg

**Affiliations:** Department of Neurology and Experimental Neurology, Charité-Universitätsmedizin Berlin, Corporate Member of Freie Universität Berlin, Humboldt-Universität Berlin, Berlin 10117, Germany; Einstein Center for Neurosciences Berlin, Charité- Universitätsmedizin Berlin, Berlin 10117, Germany; Department of Neurology and Experimental Neurology, Charité-Universitätsmedizin Berlin, Corporate Member of Freie Universität Berlin, Humboldt-Universität Berlin, Berlin 10117, Germany; Institute for Immunity, Transplantation, and Infection, Stanford University School of Medicine, Stanford, CA 94305, USA; Berlin Institute of Health at Charité, Universitätsmedizin Berlin, Berlin 10178, Germany; Berlin School of Mind and Brain, Humboldt-Universität zu Berlin, Berlin 10117, Germany; Department of Neurology, St. Josef Hospital, Ruhr University Bochum, Bochum 44791, Germany; Department of Neurology, Evangelic Hospital Lippstadt, Lippstadt 59555, Germany; French Reference Centre on Paraneoplastic Neurological Syndromes and Autoimmune Encephalitis, Hospices Civils de Lyon, Lyon 69677, France; Department of Neurology, Hospital Universitario 12 de Octubre, Madrid 28041, Spain; French Reference Centre on Paraneoplastic Neurological Syndromes and Autoimmune Encephalitis, Hospices Civils de Lyon, Lyon 69677, France; Institut MeLiS INSERM U1314/CNRS UMR 5284, Université Claude Bernard Lyon 1, 69372 Lyon, France; Department of Neurology, Erasmus University Medical Center, Rotterdam 3015GD, The Netherlands; Lyon ALS Reference Center, Hôpital Neurologique Pierre Wertheimer, Hospices Civils de Lyon, Université de Lyon, Bron 69677, France; Department of Neurology, Johannes Kepler University Linz, Linz 4020, Austria; Department of Laboratory Medicine and Pathology, Mayo Clinic, Rochester, MN 55905, USA; Department of Neurology, Mayo Clinic, Rochester, MN 55905, USA; Center for Multiple Sclerosis and Autoimmune Neurology, Mayo Clinic, Rochester, MN 55905, USA; Department of Neurology, Medical Faculty and University Hospital, Heinrich-Heine-University Düsseldorf, Düsseldorf 40225, Germany; Department of Neurology, Center for Neurology and Neuropsychiatry, LVR-Klinikum, Heinrich-Heine-University Düsseldorf, Düsseldorf 40629, Germany; Department of Neurology, Martha-Maria Hospital Halle, Halle 06120, Germany; Department of Neurology with Institute of Translational Neurology, University of Münster, Münster 48149, Germany; Institute of Clinical Chemistry, University Hospital Schleswig-Holstein, Kiel 24105, Germany; Department of Neurology, Erasmus University Medical Center, Rotterdam 3015GD, The Netherlands; Department of Neurology, Erasmus University Medical Center, Rotterdam 3015GD, The Netherlands; Laboratory of Medical Immunology, Department of Immunology, Erasmus University Medical Center, University Medical Center, Rotterdam 3015GD, The Netherlands; Section Translational Neuroimmunology, Jena University Hospital, Jena 07747, Germany; Department of Neurology, University Hospital Ulm, Ulm 89081, Germany; Department of Pediatrics, University Hospital Schleswig-Holstein, Lübeck 23562, Germany; Department of Neurology, Medical Faculty and University Hospital, Heinrich-Heine-University Düsseldorf, Düsseldorf 40225, Germany; Institute of Clinical Chemistry, University Hospital Schleswig-Holstein, Kiel 24105, Germany; Department of Neurology, University Hospital Schleswig-Holstein, Kiel 24105, Germany; Department of Neurology, Odense University Hospital, Odense 5000, Denmark; Department of Clinical Research, University of Southern Denmark, Odense 5230, Denmark; Department of Neurology, Mayo Clinic, Rochester, MN 55905, USA; Center for Multiple Sclerosis and Autoimmune Neurology, Mayo Clinic, Rochester, MN 55905, USA; Neurology Service, Hospital Clínic of Barcelona, Biomedical Research Institute (IDIBAPS), Barcelona 08036, Spain; Neuroimmunology Program, Fundació de Recerca Clínic Barcelona-Institut D’Investigacions Biomèdiques August Pi I Sunyer (FCRB-IDIBAPS) - Caixa Research Intitute (CRI), Barcelona 08036, Spain; Universitat de Barcelona, Barcelona 08036, Spain; Centro de Investigación Biomédica en Red, Enfermedades Raras (CIBERER), Madrid 28029, Spain; Neurology Service, Hospital Clínic of Barcelona, Biomedical Research Institute (IDIBAPS), Barcelona 08036, Spain; Neurology Service, Hospital Clínic of Barcelona, Biomedical Research Institute (IDIBAPS), Barcelona 08036, Spain; Neurology Service, Hospital Clínic of Barcelona, Biomedical Research Institute (IDIBAPS), Barcelona 08036, Spain; Neuroimmunology Program, Fundació de Recerca Clínic Barcelona-Institut D’Investigacions Biomèdiques August Pi I Sunyer (FCRB-IDIBAPS) - Caixa Research Intitute (CRI), Barcelona 08036, Spain; Department of Neurology, University Pennsylvania, Philadelphia, PA 19104, USA; Department of Neurology and Experimental Neurology, Charité-Universitätsmedizin Berlin, Corporate Member of Freie Universität Berlin, Humboldt-Universität Berlin, Berlin 10117, Germany; German Center for Neurodegenerative Diseases (DZNE) Berlin, Berlin 10117, Germany; Division of Neuropathology and Neurochemistry, Department of Neurology, Medical University of Vienna, Vienna 1090, Austria; Comprehensive Center for Clinical Neurosciences and Mental Health, Medical University of Vienna, Vienna 1090, Austria; Department of Neurology, University Hospital Zurich, Zurich 8091, Switzerland; Institute of Experimental Immunology, University of Zurich, Zurich 8057, Switzerland; Department of Laboratory Medicine and Pathology, Mayo Clinic, Rochester, MN 55905, USA; Department of Neurology, University Hospital Ulm, Ulm 89081, Germany; Department of Neurology, Mayo Clinic, Jacksonville, FL 32224, USA; Department of Neurosciences, Mayo Clinic, Jacksonville, FL 32224, USA; Oxford Autoimmune Neurology Group, Nuffield Department of Clinical Neurosciences, 9 University of Oxford, Oxford OX3 9DU, UK; Stanford Center for Sleep Sciences and Medicine, Stanford University School of Medicine, Stanford, CA 94305, USA; Department of Neurology, Erasmus University Medical Center, Rotterdam 3015GD, The Netherlands; Department of Neurology, St. Josef Hospital, Ruhr University Bochum, Bochum 44791, Germany; French Reference Centre on Paraneoplastic Neurological Syndromes and Autoimmune Encephalitis, Hospices Civils de Lyon, Lyon 69677, France; Department of Neurology and Experimental Neurology, Charité-Universitätsmedizin Berlin, Corporate Member of Freie Universität Berlin, Humboldt-Universität Berlin, Berlin 10117, Germany; Berlin Center for Advanced Neuroimaging, Charité- Universitätsmedizin Berlin, Corporate Member of Freie Universität Berlin and Humboldt-Universität zu Berlin, Berlin 10117, Germany

**Keywords:** autoimmune encephalitis, IgLON5, MRI, atrophy

## Abstract

Anti-IgLON5 disease is an autoimmune encephalitis that presents with a heterogenous clinical phenotype, including sleep disorders, movement abnormalities and bulbar involvement. It is characterized by autoantibodies against IgLON5, 85% association with HLA-DQB1*05:∼ and a brainstem-dominant tauopathy. Cellular and murine models report pathogenic effects of the autoantibodies, and neurodegenerative factors suggest progressive atrophy as a common sequela. However, evidence from *in vivo* patient data and long-term follow-up is limited, and the degree of progression remains elusive.

In this multicentre study, clinical and brain MRI data were collected from 127 patients across 12 countries to investigate the relationships between clinical presentations and the development of distinct brain atrophy patterns. Our data show that most patients develop a complex multisystem phenotype as the disease progresses; however, neuromuscular manifestations rarely emerge at later disease stages. By comparison to healthy controls, this disease presents with severe substructure-specific atrophy, especially affecting the hypothalamus, brainstem, accumbens and basal ganglia, which, in age-independent analyses, show significant ventricular enlargement and also suggest progression of brainstem atrophy over the disease course. Moreover, the focality of atrophy was functionally linked to specific symptoms, with more severe involvement of the basal ganglia in patients with movement disorders, and greater atrophy in the hippocampus and thalamus in patients with cognitive impairment.

Taken together, our results provide evidence of distinct atrophy patterns in anti-IgLON5 disease, which closely mirror sites of pathophysiologic processes, including autoantibody binding and tau deposition. Our data emphasize the brainstem as the pathophysiological hub of the disease and provide normative data for the incorporation of atrophy measurements into routine clinical assessments and future treatment studies to monitor disease trajectory and evaluate future treatment strategies.

## Teaser

Yogeshwar *et al.* present clinical and brain MRI data from 127 patients with anti-IgLON5 disease across 12 countries. They demonstrate brain atrophy patterns that closely mirror sites of autoantibody binding and tau accumulation, and show that the location of atrophy is functionally linked to specific symptoms.

## Introduction

Anti-IgLON5 disease predominantly affects patients above the age of 60 years, showing a heterogeneous clinical presentation^1^ involving sleep, bulbar, movement, neuromuscular and cognitive manifestations.^2,3^ The majority of patients present with a multisystem phenotype.^4^ Response to immunotherapy varies depending on disease stage, with only half of those treated showing an improvement.^4,5^ The key autoimmune hallmarks of the disease are autoantibodies targeting IgLON5 that disrupt cytoskeletal organization, reduce synaptic protein content, contribute to an inflammatory response and lead to both phosphorylated-tau accumulation and cell death.^6-9^ Moreover, the disease is 85% associated with HLA-DQA1*01:05∼DQB1*05:01, HLA-DQA1*01:01∼DQB1*05:01 and HLA-DQA1*01:04∼DQB1*05:03 haplotypes^10^ (denoted as HLA-DQB1*05:∼). These encode HLA molecules that bind IgLON5-derived peptides, evoking elevated CD4^+^ T cell reactivities that likely contribute to the autoimmune response initiation.^10^

Neuropathological and PET studies in patients with anti-IgLON5 disease highlight the presence of inflammation, neuronal loss and hyperphosphorylated tau deposition, with a preferential involvement of the brainstem and hypothalamus.^1,11-14^ While there is evidence of cellular dysfunction and atrophy in murine models,^6-9,15-17^  *in vivo* evidence from anti-IgLON5 disease patients remains scarce, with only individual MRI studies indicating atrophy of the cerebellum, cortex, midbrain and brainstem.^18-22^

Understanding atrophy patterns may help better understand the pathophysiological mechanisms of neurological diseases and their progression, and provide quantitative measures to evaluate the efficacy of therapeutic interventions.^23^ For example, studies on anti-NMDA receptor (NMDAR) encephalitis^24^ and anti-LGI1 encephalitis^25^ identified links between cognitive deficits and hippocampal structural damage that could partially be prevented by early immunotherapy in the latter disease.^26^ In anti-IgLON5 disease, it remains elusive whether atrophy is frequent and whether it associates with genotype, treatment or distinct clinical manifestations.

Here, we investigate brain atrophy patterns in anti-IgLON5 disease using MRI in the largest established cohort to date. We assess the association between the development and evolution of atrophy and distinct clinical manifestations, HLA-predispositions, disease progression and immunotherapies. This approach provides insights into the anatomical pathophysiology underlying this condition, as well as generating quantitative measurements of value in future clinical trials.

## Materials and methods

### Collection of patient MRI and clinical data

**Figure 1:**
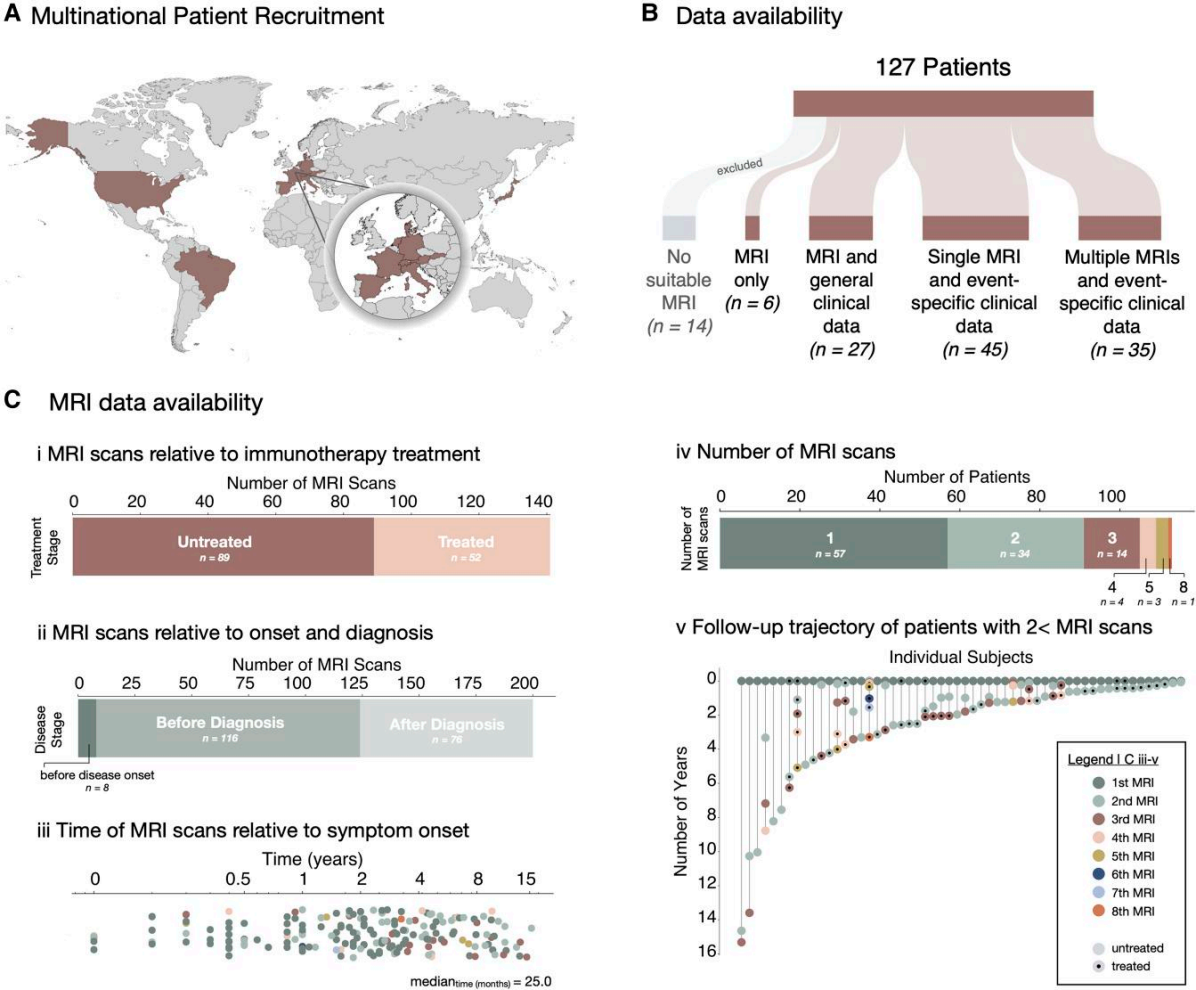
**Data collection from anti-IgLON5 patients.** (**A**) Retrospective patient data were collected from 12 countries: Germany (*n* = 39), France (*n* = 37), USA (*n* = 15), the Netherlands (*n* = 13), Spain (*n* = 6), Switzerland (*n* = 5), Austria (*n* = 4), Italy (*n* = 4), Brazil (*n* = 1), Slovakia (*n* = 1), Japan (*n* = 1) and Denmark (*n* = 1). (**B**) Availability of clinical and MRI data for all patients. [**C**(**i**–**v**)] MRI data availability. (**i**) Number of MRI scans available relative to immunotherapy treatment. (**ii**) Number of MRI scans available relative to disease onset and diagnosis. (**iii**) Time of MRI scans relative to disease onset. (**iv**) Number of MRI scans available across patients. (**v**) Follow-up trajectory of patients with two or more MRI scans and treatment status at time of MRI.

A literature search was conducted to identify patients with anti-IgLON5 disease, and corresponding authors and centres were contacted to request patient data. A total of 257 retrospective brain MRI datasets were collected from a multicentre cohort of 127 patients with anti-IgLON5 disease (Fig. 1). Patients were recruited from Germany (*n* = 39), France (*n* = 37), USA *(n* = 15), the Netherlands (*n* = 13), Spain (*n* = 6), Switzerland (*n* = 5), Austria (*n* = 4), Italy (*n* = 4), Brazil (*n* = 1), Slovakia (*n* = 1), Japan (*n* = 1) and Denmark (*n* = 1) (Fig. 1A). All patients tested positive for anti-IgLON5 antibodies in serum and/or CSF: 98% (113/115) tested positive in serum; 88% (98/112) in CSF; and 84% (84/100) in both. The study was conducted in accordance with the Declaration of Helsinki, and all enrolled patients or their legal representatives provided written informed consent.

A total of 51 scans were excluded from analyses due to a lack of compatible MRI sequences for subcortical segmentation (*n* = 49), excessive head movement (*n* = 1) or brain tumour (*n* = 1), resulting in 206 MRIs from 113 patients being carried forward for further analyses (Fig. 1B).

One MRI study was available for 50.4% (57/113) of patients, and 49.6% (56/113) of patients had MRI data from two or more follow-ups [for details, see Fig. 1C(iv)], with varying durations between scans [Fig. 1C(v)]. Scans were available from various disease stages [Fig. 1C(ii)] and the median time between onset of the disease and the first brain MRI study was 25 [interquartile range (IQR): 9.0–53.5] months [Fig. 1C(iii)].

**Figure 2:**
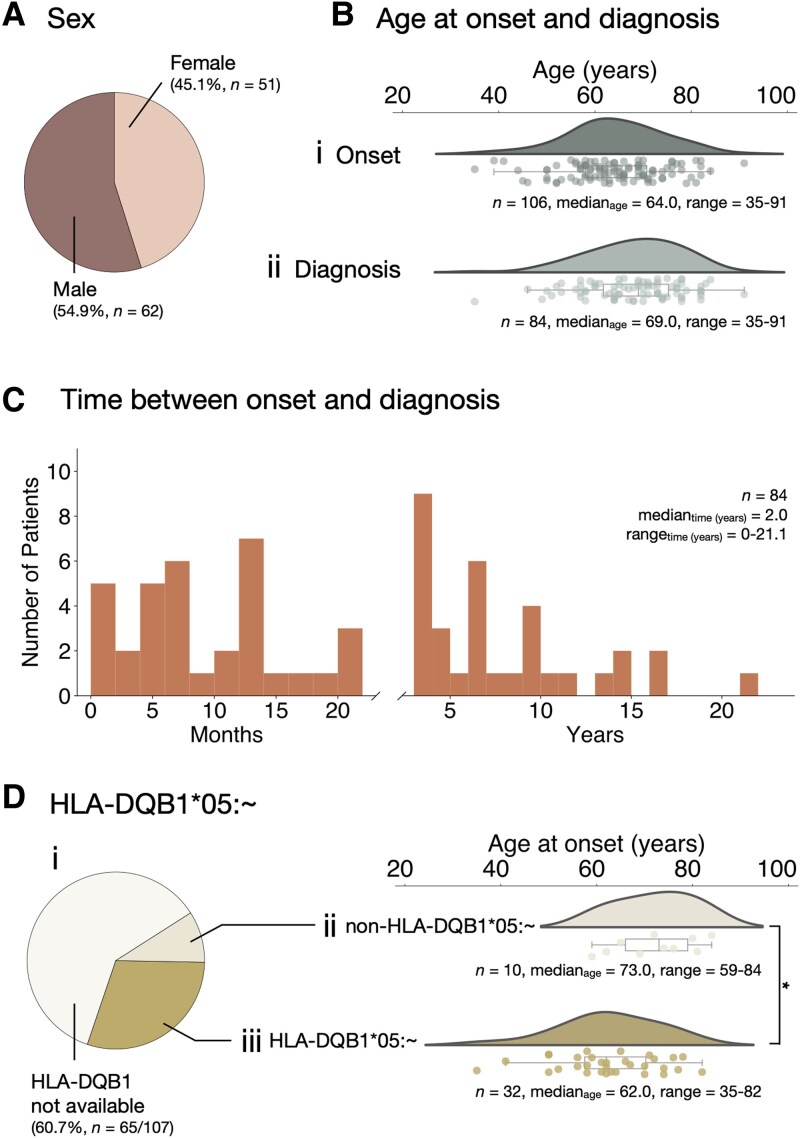
**Demographic presentation and HLA of anti-IgLON5 patients.** (**A**) Sex distribution of patients. [**B**(**i** and **ii**)] Age of patients at disease (**i**) onset and (**ii**) diagnosis. (**C**) Time between disease onset and diagnosis. [**D**(**i**–**iii**)] HLA-DQB1*05:∼ status is known from 39.3% (42/107) subjects. (**i**) Carrier frequency highlights predominance of HLA-DQB1*05:∼ over non-HLA-DQB1*05:∼. (**ii**) Age at disease onset of non-HLA-DQB1*05:∼-carriers and (**iii**) HLA-DQB1*05:∼-carriers highlights significantly younger age at disease onset in HLA-DQB1*05:∼-carriers versus non-HLA-DQB1*05:∼-carriers. *P < 0.05.

**Figure 3:**
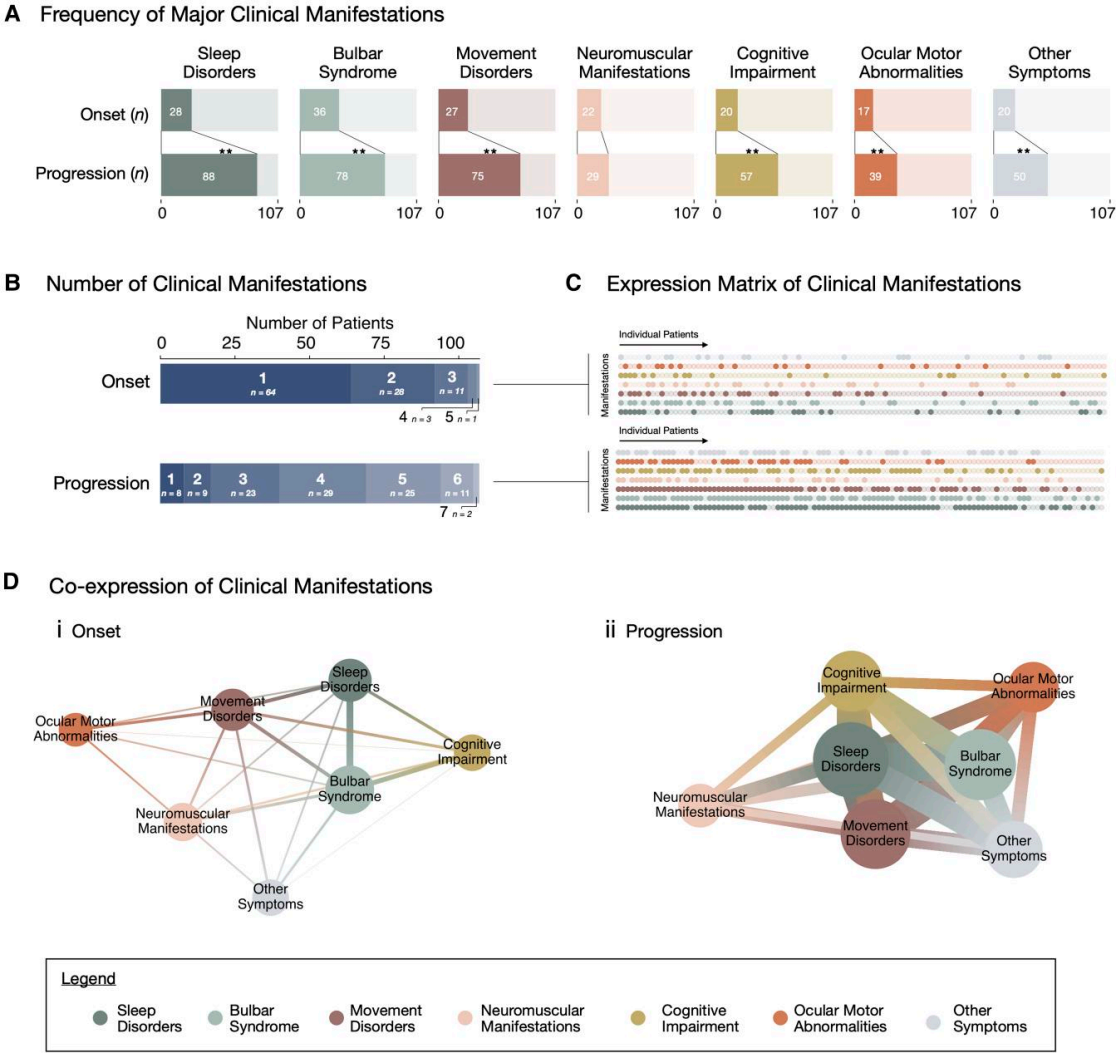
**Clinical presentation of anti-IgLON5 patients.** (**A**) Frequency of major clinical manifestations (sleep disorder, bulbar syndrome, movement disorders, neuromuscular manifestations, cognitive impairment, ocular motor abnormalities and/or other symptoms) at symptom onset and disease progression. All symptoms except for neuromuscular manifestations significantly increase in presentation over disease progression compared to symptom onset. (**B**) Number of major clinical manifestations at symptom onset and disease progression highlight that the majority of patients experience only a single manifestation at onset, however, develop a multisystem phenotype as the disease progresses. (**C**) Presentation of major clinical manifestations by each patient at symptom onset and disease progression (light hue = manifestation not present; dark hue = manifestation present). [**D**(**i** and **ii**)] Co-occurrence of major clinical manifestations with network analysis highlighting their interconnections at symptom (**i**) onset and (**ii**) progression. The size of centroids highlights the frequency of each manifestation, the connecting lines which manifestations co-occur, and the thickness of the connecting lines what the frequency of co-occurrence is. The frequency and co-occurrence of all manifestations is increased at disease progression compared to onset. **P < 0.01.

Clinical data were available for 107/113 (94.7%) patients (Figs 1B, 2 and 3) and were collected from medical records and physician questionnaires.^4^ Patients were classified according to the major anti-IgLON5 disease clinical manifestations, i.e. sleep disorder (parasomnia, stridor, sleep apnoea), bulbar syndrome (dysarthria, dysphagia, vocal cord paralysis), movement disorders (chorea, bradykinesia, cerebellar ataxia, dystonia, rigidity, tremor, myoclonus), neuromuscular manifestations (weakness, fasciculations), cognitive impairment (memory deficits, executive dysfunction), ocular motor abnormalities (gaze palsy, ptosis) and/or other symptoms (neuropsychiatric symptoms, autonomic dysfunction, vestibular dysfunction, epileptic seizures), as previously reported.^3,18,27,28^ Onset symptoms were defined as the initial clinical manifestations at symptom onset, whereas any symptoms that patients developed later over the disease course were classified as progression symptoms. Clinical data at the time of individual brain MRI events were available for 74.8% (80/107) of patients (Fig. 1B), whereby 63.1% (89/141) of the scans were recorded while patients were (still) untreated and 36.9% (52/141) after patients had received immunotherapy [Fig. 1C(i)].

### Control participants

Neuroimaging data for cognitively normal control participants were obtained from the Alzheimer’s Disease Neuroimaging Initiative (ADNI) database (adni.loni.usc.edu) (*n* = 957) and the Open Access Series of Imaging Studies^29^ (OASIS, http://www.oasis-brains.org) (*n* = 423) (Supplementary Fig. 1).

### Preprocessing and volumetry segmentation of MRI data

Volumetric segmentation was performed with the FreeSurfer image analysis suite^30,31^ (version 7.3.2). This processing stream includes steps for motion correction, intensity normalization, skull stripping, and the labelling of cortical gyri and sulci prior to performing automated cortical and subcortical segmentation, surface reconstruction and volumetric analysis. For the analysis of disease-progression dependent changes in volumetry, the FreeSurfer longitudinal stream was employed.^32^ Importantly, this pipeline reduces the impact of interindividual morphological variability by employing each subject as their own control. Furthermore, the deep-learning tool HypothalamicSubunits^33^ was employed to segment the hypothalamus and attain its volumetric estimate, and the toolbox BrainstemSubstructures^34^ was used to segment the medulla oblongata, pons and midbrain. Outputs were visually inspected in FreeView. This study was hypothesis driven, focusing on investigating volumetry of a subset of subcortical structures that were previously reported to be affected by pathological alterations in anti-IgLON5 disease on the basis of neuropathological^1,11,13^ and PET^12,14^ studies, namely hypothalamus, accumbens, brainstem, putamen, caudate, pallidum, hippocampus, thalamus and amygdala, as well as whole brain volume and ventricular size.

### Statistical analyses

We report median and interquartile range (IQR) for continuous variables, absolute and relative frequencies for categorical variables, stratified by patient groups, where meaningful. Continuous variables are displayed using histograms, box plots and density curves, categorical variables using pie charts and stacked bar charts, and raw data using individual data-points. Co-occurrence of clinical manifestations is displayed using a network diagram.

A Mann–Whitney U-test was used to assess the difference in age at disease onset between HLA-DQB1*05:∼-carriers and non-carriers. A Wilcoxon signed-rank test was used to assess the difference in frequency of clinical manifestations at symptom onset versus progression. MRI studies of 206 control participants were age- and sex-matched to patients at a 1:1 ratio^35^ (Supplementary Fig. 1) for analyses using FreeSurfer recon-all, brainstem substructures and hypothalamus. Linear mixed-effects models (LMM) were fitted to analyse trends and differences in atrophy patterns.^36,37^ The effects of sex, age at visit (fixed effects) and repeat measures in the same individual (random intercept) were controlled for in all analyses. Especially in longitudinal studies, controlling for age allowed for the determination of age-independent, disease-progression-dependent volume loss. Moreover, individual analyses were further controlled for the effects of HLA, disease progression, distinct clinical manifestations and treatment. Details on the exact parameter specifications (fixed effects, interaction terms, random intercepts, detailed descriptions of datasets used) of all LMM analyses are provided in Supplementary Table 1. Where multiple comparisons across different brain structures were performed, Benjamini-Hochberg (BH) adjusted *P*-values are reported. *P*-values are explicitly marked as either unadjusted (*P*_unadj_) or adjusted (*P*_adj_). All analyses were carried out in Python and R.

### Visualizations

Visualizations for all figures were prepared using Python, R, Keynote, SankeyMatic, WorldMap MapChart and FreeSurfer. Data for the visualization of IgLON5 expression (in the ‘Anti-IgLON5 disease presents with extensive and substructure-specific atrophy’ section) were obtained from the Human Protein Atlas^38^ (proteinatlas.org, ENSG00000142549). Data for the visualization of IgG deposition were obtained from Berger-Sieczkowski *et al.*^13^ and data for the visualization of tau pathology from Gelpi *et al.*,^11,39^ Schöberl *et al.*,^12^ Theis *et al.*,^14^ Berger-Sieczkowski *et al.*^13^ and Fan *et al*.^40^

## Results

### Patient demographics and HLA characteristics

In our study, 54.9% (*n* = 62/113) of anti-IgLON5 disease patients were male (Fig. 2A). The median age of symptom onset was 64.0 (IQR: 58.0–70.8) years [Fig. 2B(i)] and 69.0 (IQR: 61.8–75.3) years [Fig. 2B(ii)] at diagnosis. Hence, the time lag between symptom onset and diagnosis was a mean of 3.8 years (median of 24.5 months, IQR = 11.8–56.5 months) (Fig. 2C). *HLA-DQB1* status was available for 42 patients and showed that 76.2% (32/42 patients) carried HLA-DQB1*05:∼ [Fig. 2D(i)], which was also associated with a significantly younger age at disease onset (mean difference = 9.7 years, *P* = 0.014) compared to non-HLA-DQB1*05:∼-carriers [Fig. 2D(ii and iii)].

### Clinical evolution in anti-IgLON5 disease

In descending order of frequencies, the most prominent manifestations at onset were bulbar syndrome (33.6%, 36/107 patients), sleep disorders (26.2%, 28/107) and movement disorders (25.2%, 27/107) (Fig. 3A). The number and frequency of symptoms increased during disease progression in the majority of patients, whereby sleep disorders were then present in 82.2% (88/107) of patients, followed by movement disorders (70.1%, 75/107) and bulbar syndrome (72.9%, 78/107). Interestingly, the frequency of neuromuscular manifestations did not change significantly between symptom onset (20.6%) and later disease stages (27.1%) (Fig. 3A). In line with these symptom trajectories, 59.8% (64/107) of patients experienced symptoms of only a single major clinical manifestation at initial disease presentation (Fig. 3B and C, top), with few reporting concurrent onset of multiple symptoms. Subsequently, the number of symptoms increased over the course of disease progression, with 92.5% (99/107) experiencing symptoms of two or more major clinical manifestations (Fig. 3B and C, bottom). Furthermore, the most common co-occurrence of features at initial disease presentation was a bulbar syndrome with sleep and cognitive impairments [Fig. 3C and D(i) and Supplementary Table 2]. During subsequent progression, more co-occurrence of all features emerged, in line with the evolution of a multisystem phenotype^4^ [Fig. 3D(ii) and Supplementary Table 3].

### Anti-IgLON5 disease presents with extensive and substructure-specific atrophy

**Figure 4:**
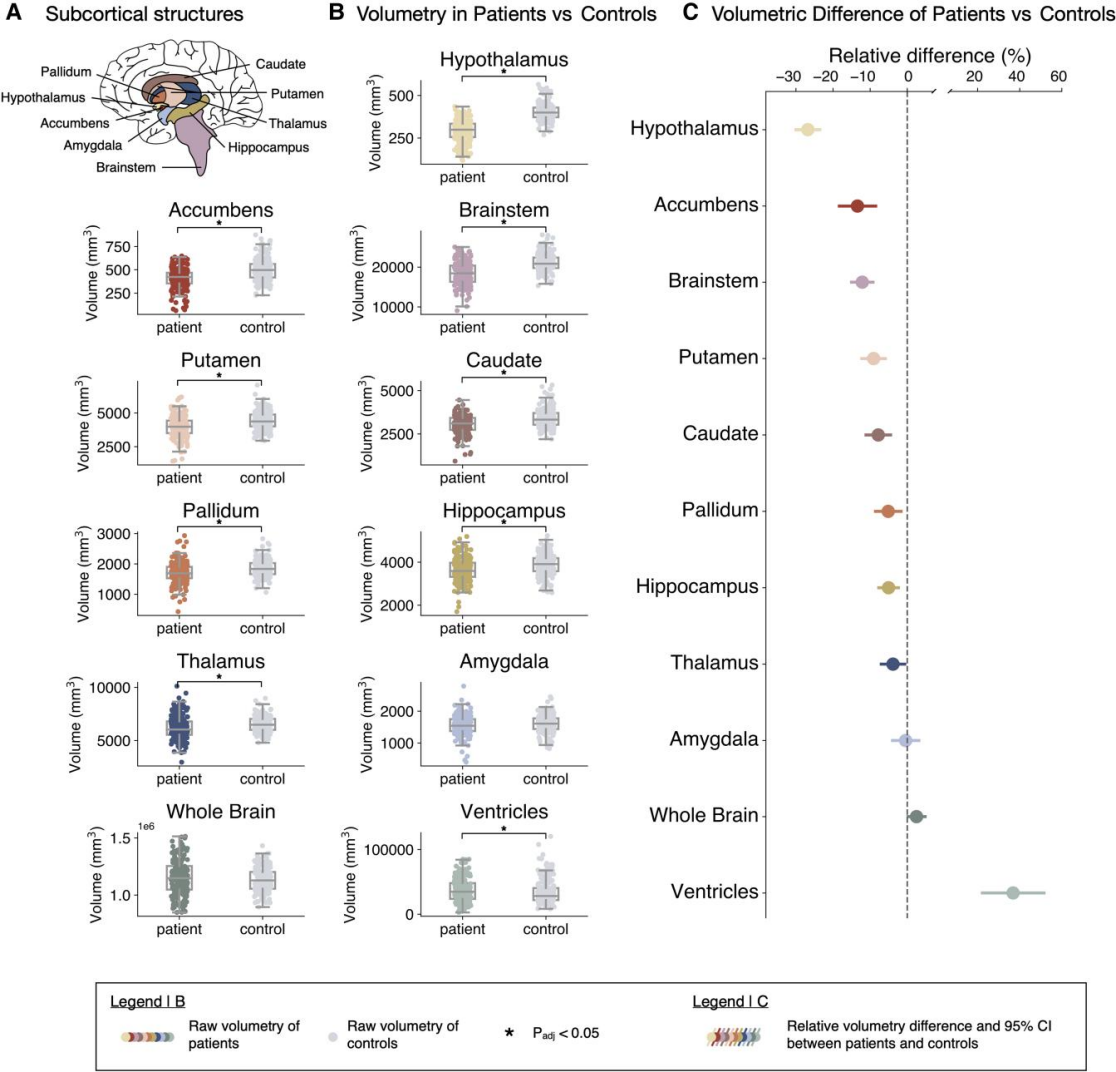
**Cross-sectional volumetry of anti-IgLON5 patients.** (**A**) Anatomy of subcortical structures in their respective colour-coding that is carried forward in the remaining figure. (**B**) Region of interest (ROI) volumetry of patients (coloured) and matched controls (grey) shows significantly smaller volumetry in patients compared to controls in the hypothalamus, accumbens, brainstem, putamen, caudate, pallidum, hippocampus, thalamus and enlarged ventricles. (**C**) Relative difference and 95% confidence interval (CI, in %) in ROI volumetry between patients and matched controls shown in **B**. *P_adj_ < 0.05. For details on parameters included in analyses, see Supplementary Table 1.

To assess disease-dependent atrophy patterns, brain volumetry was compared between patients and matched controls (Supplementary Fig. 1). These analyses revealed atrophy of several subcortical structures in patients compared to controls, most prominently hypothalamus (volume −26.8%; 95% CI: −30.0%, −23.6%; *P*_unadj_ = 5.0 × 10^−39^, *P*_adj_ = 5.5 × 10^−38^), accumbens (volume −13.4% 95% CI: −18.3%, −8.5%, *P*_unadj_ = 1.9 × 10^−7^, *P*_adj_ = 5.2 × 10^−7^), brainstem (−12.1%; CI: −15.0%, −9.2%; *P*_unadj_ = 2.2 × 10^−14^, *P*_adj_ = 1.2 × 10^−13^), putamen (−9.1%; CI: −12.2%, −5.9%; *P*_unadj_ = 6.1 × 10^−8^, *P*_adj_ = 2.2 × 10^−7^), caudate (−7.8%; CI: −11.1%, −4.5%; *P*_unadj_ = 6.0 × 10^−6^, *P*_adj_ = 1.1 × 10^−5^), pallidum (−5.1%; CI: −8.6%, −1.7%; *P*_unadj_ = 3.9 × 10^−3^, *P*_adj_ = 5.3 × 10^−3^), hippocampus (−5.0%; CI: −7.7%, −2.4%; *P*_unadj_ = 2.3 × 10^−4^, *P*_adj_ = 3.7 × 10^−4^) and thalamus (−3.8%; CI: −7.0%, −0.7%; *P*_unadj_ = 1.8 × 10^−2^, *P*_adj_ = 2.2 × 10^−2^) (Fig. 4). Further segmentation of the brainstem revealed the most prominent atrophy in the medulla (volume −20.9%; 95% CI: −23.7%, −18.1%; *P*_unadj_ = 1.5 × 10^−33^, *P*_adj_ = 4.5 × 10^−33^), followed by the pons (volume −8.0%; 95% CI: −10.8%, −5.2%; *P*_unadj_ = 6.3 × 10^−8^, *P*_adj_ = 9.5 × 10^−8^) and midbrain (volume −2.7%; 95% CI: −5.2%, −0.1%; *P*_unadj_ = 4.3 × 10^−2^, *P*_adj_ = 4.3 × 10^−2^) (Supplementary Fig. 2). No significant volume reduction was observed for the amygdala. Atrophy in the patient cohort was further associated with significantly enlarged ventricles (36.7%; CI: 22.3%, 51.5%; *P*_unadj_ = 1.5 × 10^−6^, *P*_adj_ = 3.2 × 10^−6^), but not whole brain volume reduction, consistent with a more focal atrophy pattern.

### Disease progression exacerbates the evolution of selected substructure-specific atrophy

**Figure 5:**
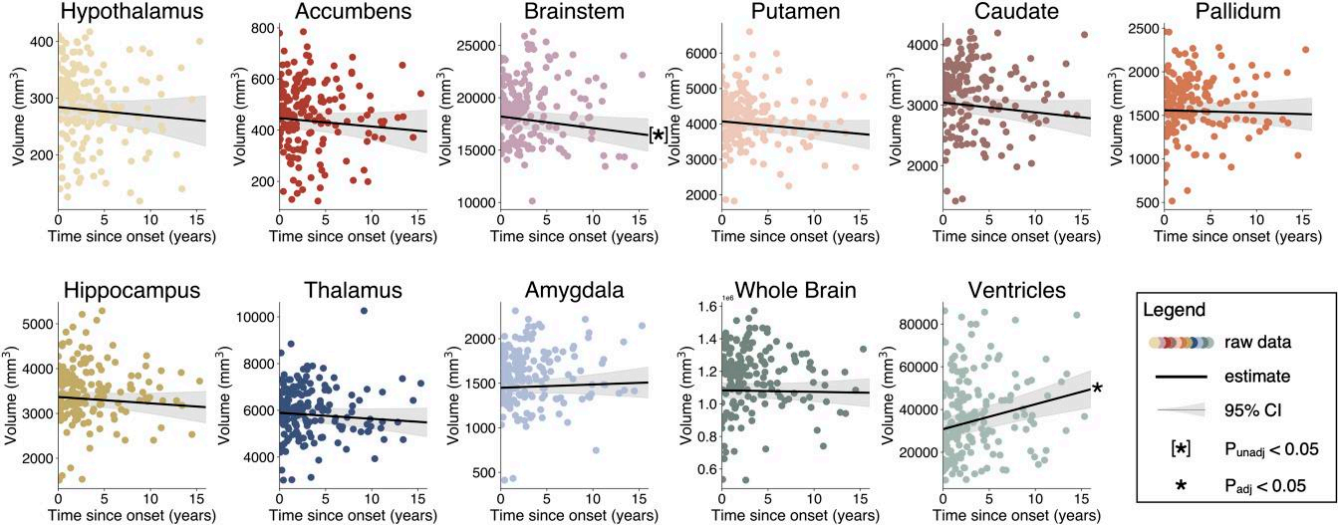
**Longitudinal changes in volumetry.** (**A**) Volumetry of regions of interest (for anatomy, see Fig. 4A) over disease course (= time since disease onset) in years. Scatter plots show raw data and the black lines show the estimate + 95% confidence interval (shaded area) of change in volumetry over the disease course. A trend towards disease progression dependent reduction in the brainstem (*P*_unadj_ < 0.05) is visible, as well as significant enlargement of the ventricles (*P*_adj_ < 0.05). *P_adj_ < 0.05, ^[*]^*P*_unadj_ < 0.05. For details on parameters included in analyses, see Supplementary Table 1.

Next, clinical and pathophysiological factors that might underlie substructure-specific atrophy were investigated. First, the association between disease stage and atrophy was examined. To accurately trace disease progression-dependent changes in volumetry, subcortical segmentation of subjects with multiple MRI studies was conducted using the FreeSurfer longitudinal pipeline, which allows for accurate interindividual tracing of longitudinal volumetric changes.^32^ Investigating age-independent, disease-progression-dependent volume loss (Supplementary Table 1), increasing ventricle volume size (2.8%; *P*_unadj_ = 1.9 × 10^−5^, *P*_adj_ = 2.1 × 10^−4^) was observed, together with a trend for progressive atrophy in the brainstem (annual volume change, −0.5%; *P*_unadj_ = 0.03, *P*_adj_ = 0.14) (Fig. 5). In addition, hypothalamus (−0.5%; *P*_unadj_ = 0.35, *P*_adj_ = 0.48), putamen (−0.4%; *P*_unadj_ = 0.11, *P*_adj_ = 0.36), caudate (−0.5%; *P*_unadj_ = 0.12, *P*_adj_ = 0.36), thalamus (−0.3%; *P*_unadj_ = 0.21, *P*_adj_ = 0.45), hippocampus (−0.3%; *P*_unadj_ = 0.26, *P*_adj_ = 0.45) and accumbens (−0.8%; *P*_unadj_ = 0.29, *P*_adj_ = 0.45) also showed volume reductions with increasing disease duration that were less pronounced and did not reach significance.

### Atrophy predilection sites mirror nuclei of symptomatic manifestations

**Figure 6:**
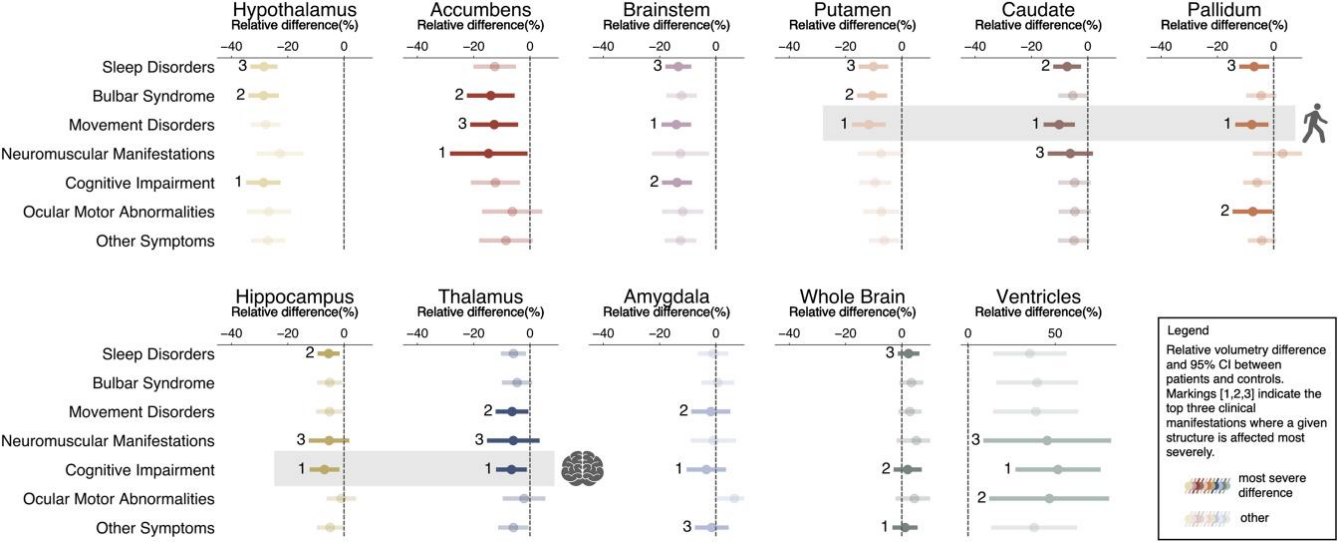
**Relationship between clinical manifestations and volumetry.** (**A**) Relative difference and 95% confidence interval (CI, in %) in region of interest volumetry (for anatomy, see Fig. 4A) between patients presenting with specific clinical manifestation at the time of the MRI (Supplementary Tables 1–3) and matched controls. Numbers (1, 2, 3) indicate which manifestations are affected by the most severe difference in volumetry compared to controls. Grey background of putamen, caudate and pallidum show atrophy is severest in patients suffering from movement disorders. Grey background of hippocampus and thalamus show atrophy is severest in patients suffering from cognitive impairment. For details on parameters included in analyses, see Supplementary Table 1.

Next, due to the heterogenous symptomatic presentation of anti-IgLON5 disease, associations between different clinical manifestations and substructure-specific atrophy were examined (Fig. 3). Extensive substructure-specific atrophy across all clinical subtypes was confirmed (Fig. 6). Especially hypothalamus, accumbens, brainstem, putamen and caudate revealed consistent and severe atrophy across all clinical manifestations, while ventricular enlargement was also observed consistently across all clinical manifestations. However, the degree of regional-specific atrophy varied depending on the clinical presentation of patients (Fig. 6). As such, the basal ganglia, and especially caudate, show a trend for being more severely atrophied in patients presenting with movement disorders (Fig. 6, top), whereas thalamus and hippocampus were more affected in patients with cognitive impairment (Fig. 6, bottom). Lastly, rarer and less specific clinical manifestations involving ocular-motor abnormalities and other symptoms generally present with milder and less focal atrophy. Neither increased co-occurrence of multiple different clinical manifestations (Fig. 3D and Supplementary Fig. 3), nor disease progression within clinical subgroups (Supplementary Fig. 4) associated with distinct atrophy patterns. Given the strong (∼85%) association of anti-IgLON5 disease with HLA-DQB1*05:∼,^10^ the effect of HLA-DQB1*05:∼ on atrophy development and evolution was assessed but showed no consistent effects (Supplementary Fig. 5). Furthermore, an exploratory analysis revealed no impact of immunotherapy on atrophy (Supplementary Table 1 and Supplementary Fig. 6).

## Discussion

**Figure 7:**
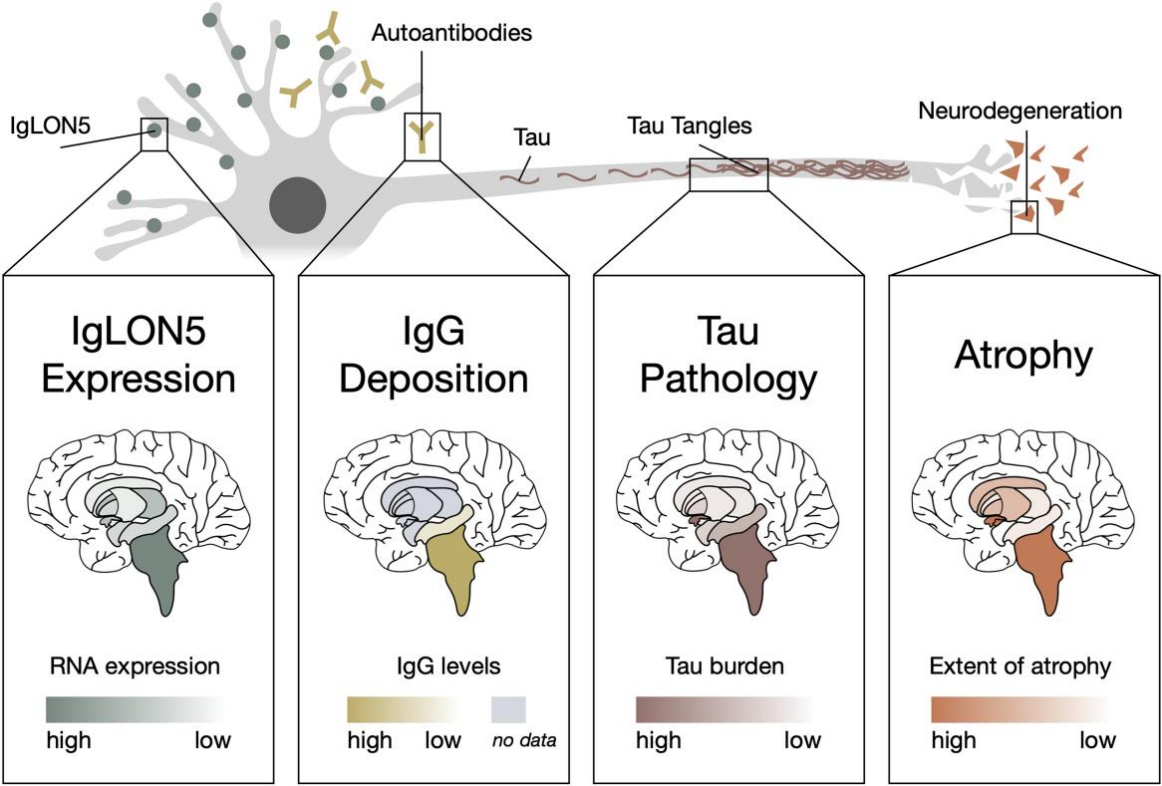
**From autoimmunity to neurodegeneration.**  *Top*: Suggested molecular pathophysiology of anti-IgLON5 disease. IgLON5 is expressed extra-cellularly on neurons and targeted by IgLON5 autoantibodies. Intracellularly, hyperphosphorylated tau accumulates, ultimately forming tau tangles and leading to neurodegeneration. *Bottom*: Substructures investigated in this study are shown. IgLON5 expression: Expression of IgLON5 RNA according to the Human Protein Atlas^38^ (proteinatlas.org, ENSG00000142549). IgG deposition: Deposition of IgG1 and IgG4 antibodies is shown according to Berger-Sieczkowski *et al.*^13^ Tau pathology: Distribution of tau pathology is shown according to Gelpi *et al.*,^11,39^ Schöberl *et al.*,^12^ Theis *et al.*,^14^ Berger-Sieczkowski *et al.*^13^ and Fan *et al.*^40^ Atrophy: Predilection sites of atrophy demonstrated in this study are shown. For an anatomical legend of subcortical structures, refer to Fig. 4A.

This study reports the largest cohort of anti-IgLON5 disease patients to date and presents evidence of a specific brain atrophy pattern. Our results identify the brainstem as the key predilection site of atrophy, further to substructure-specific atrophy affecting the hypothalamus, accumbens, putamen, caudate, pallidum, hippocampus and thalamus, as well as increased ventricle size. Remarkably, this atrophy pattern closely mirrors IgLON5 expression,^38^ autoantibody deposition^13^ and sites of tau accumulation (Fig. 7).^1,11-14,39,40^ Moreover, ventricular enlargement and, to a milder degree, brainstem atrophy, exacerbate with disease progression. Lastly, while substructure-specific atrophy presents across all clinical subtypes, basal ganglia show a trend towards being more severely affected in patients presenting with movement disorders, and thalamus and hippocampus in patients with cognitive impairment. Taken together, our findings provide important insights into atrophy patterns and evolution in anti-IgLON5 disease, bridging *in vivo* patient data with neuropathological and molecular studies, and thus have relevant implications for the pathophysiological understanding, diagnosis and treatment monitoring of anti-IgLON5 disease.

The demographic and clinical presentations of patients in our study corroborate several previous observations,^2,4,10^ specifically: (i) a late age at disease onset; (ii) substantial time lag between symptom onset and disease diagnosis; and (iii) overrepresentation of HLA-DQB1*05:∼ in patients that is associated with a shift in age at disease onset. Moreover, we observed a shift from an oligosymptomatic presentation at disease onset to a multisystem phenotype during later disease stages, with an accordingly marked increase in the prevalence of most symptoms. Interestingly, neuromuscular symptoms were an exception and remained at a relatively modest level, potentially suggesting a distinct disease mechanism or subtype within the broader condition. Our findings on the clinical presentation of patients and the co-occurrence of different manifestations have important implications, prompting consideration of anti-IgLON5 disease and potential testing for IgLON5 autoantibodies. Indeed, a recent study^41^ that systematically screened plasma of subjects with idiopathic/isolated REM sleep behaviour disorder (iRBD) identified that 3/339 cases presented with IgLON5 autoantibodies. Moreover, findings from Grüter *et al*.^4^ revealed that a median of four physicians and frequent initial misdiagnoses are encountered by patients prior to the correct clinical diagnosis, while our study observed an average delay of 3.8 years between onset and diagnosis. These findings highlight the need to raise awareness of anti-IgLON5 disease and its clinical presentation to facilitate early, accurate diagnosis, enabling timely treatment and improved outcomes.

Our MRI analyses identified a robust and specific atrophy pattern in patients with anti-IgLON5 disease. Atrophy was shown to be substructure-specific, rather than affecting global brain volume, and to affect the hypothalamus, brainstem and accumbens most severely, followed by putamen, caudate, pallidum, hippocampus and thalamus. Remarkably, our study shows that atrophy very closely mirrors sites of: (i) high IgLON5 expression^38^; (ii) autoantibody deposition^13^; and (iii) elevated tau pathology^1,11-14,39,40^—i.e. MRI patterns reflect sites of immunopathological changes. Interestingly, this contrasts with findings in other neuroimmunological diseases, such as multiple sclerosis or anti-NMDAR encephalitis, that show weaker associations between routine brain MRI patterns and proposed pathophysiological mechanisms.^42,43^ These disorders are characterized by a clinico-radiological paradox and only advanced imaging studies identified robust associations between imaging alterations (e.g. functional network disruption or reduced microstructural integrity), suggested disease mechanisms, and clinical symptoms.^44-46^

Our results furthermore emphasize the role of the brainstem as the predominant ‘pathological hub’ in anti-IgLON5 disease. Indeed, protein expression analysis showed that IgLON5 is most abundantly expressed in the pons and medulla oblongata,^38^ while IgG4 preferentially deposits at the tegmentum of the brainstem, spanning the aforementioned structures.^13^ Further, brainstem tauopathy was confirmed *in vivo* using ^18^F-PI-2620 PET,^14^ florzolotau PET^40^ and in 15/22 cases studied post-mortem.^11,13,39^ Interestingly, these studies revealed that the severity of pathology decreases along a caudo-cranial gradient from the medulla oblongata to the pons and the midbrain. Our findings reproduce the same gradient with regard to atrophy along the brainstem, whereby the medulla was affected most severely, followed by the pons and the midbrain. Furthermore, we observed an average brainstem atrophy of −12.1% in patients compared to controls; hence, our results suggest the brainstem as an *in vivo* imaging marker for tracking the progression of anti-IgLON5 disease. Taken together, the specific atrophy pattern revealed in our study therefore reflects previous observations from molecular and neuropathological studies.

The findings of our study also align with several previous cellular and murine studies that have highlighted neurodegenerative features in anti-IgLON5 disease, showing that IgLON5 autoantibodies cause a decrease in surface-expressed IgLON5 clusters, impaired cellular function, elevated phosphorylated-tau accumulation and cell death.^6-9,15-17^ Furthermore, studies of passive-transfer murine models^9,15-17^ showed that pathogenic and inflammatory effects preferentially cluster at sites of high IgLON5 expression. As such, tau accumulation was restricted to the brainstem, hippocampus, spinal cord and midbrain,^9,15,17^ and elevated astrocyte and microglial activation was reported in the hippocampus, hypothalamus, cerebellum and midbrain.^9,16^ This specificity was also confirmed in human neuropathological and PET studies, revealing an atypical 3R/4R tauopathy in the brainstem and hypothalamus, and to a lesser degree, the hippocampus, basal ganglia, thalamus and amygdala.^1,11-14,39,40^ While similar patterns in animal models and humans are thus reflected, further research is warranted on the transferability of murine studies to human data.

Given the progressive clinical course of anti-IgLON5 disease, we next investigated the longitudinal development of brain atrophy patterns. We observed an age-independent, disease progression-dependent atrophy associated with continued ventricular enlargement and a trend for aggravated brainstem atrophy. Importantly, these analyses were rigorously controlled for the effects of age and interindividual variability in subcortical volumetry, thereby ensuring a precise assessment of the impact of disease progression on the evolution of atrophy. Indeed, our findings align with several recent observations. First, molecular studies investigating the effects of IgLON5 autoantibodies showed time-dependent aggravation of pathogenic effects and cell death.^7,8^ Second, cytotoxic tau leads to cell death and can self-propagate trans-cellularly, thus autonomously driving progressive atrophy in regions of high tau deposition.^47,48^ Third, classical brainstem tauopathy remained absent in three cases with a short disease duration studied by Berger-Sieczkowski *et al.*,^13^ suggesting that neurodegenerative pathology develops in a disease progression-dependent manner. Taken together, previous molecular data and our longitudinal imaging analyses show that anti-IgLON5 disease features core hallmarks of a progressive neurodegenerative disease.

Further to the effect of disease progression, we studied potential correlations between substructure-specific atrophy and the evolution of various clinical manifestations. We found that atrophy—while present across all symptom complexes—predominantly affects structures functionally related to distinct clinical manifestations more severely. As such, putamen, caudate and pallidum—with their key role in motor control and movement coordination—tended to be more severely affected by atrophy in patients with movement disorders. These observations align with findings by Gao *et al.*,^17^ who showed a sustained decline in motor balance associated with reduced projection fibres and cell death in the basal ganglia of mice injected with IgLON5 autoantibodies. Moreover, we observed aggravated atrophy in the hippocampus and thalamus in patients with cognitive symptoms. Indeed, Ni *et al.*^16^ showed that mice injected with IgLON5-autoantibodies exhibited cognitive deficits concurrent with neurodegeneration in the CA1 region of the hippocampus, a core structure for learning and memory. Taken together, our findings align with previous observations and pathophysiological considerations, postulating that substructure-specific atrophy contributes to symptom development and specificity in anti-IgLON5 disease.

Some methodological limitations of our study must be acknowledged. First, the retrospective nature of the collection of clinical MRI data leads to an intrinsic heterogeneity in imaging and follow-up intervals between patients. Despite the application of stringent LMMs to account for variability within the dataset, residual biases may persist due to factors such as heterogeneity in scanner hardware or acquisition protocols. These confounding variables warrant careful consideration when interpreting the findings. However, this study design permitted the collection of a large and multinational patient cohort, which is quite challenging in rare diseases such as anti-IgLON5 disease. Second, our segmentation toolbox did not include the cerebellum, which may also be affected in the disease.^11,13,39,40^ Future studies should also characterize atrophy of the cerebellum and other structures that are not predominantly affected by pathology (including neocortex) to substantiate whether atrophy follows sites of dominant autoimmune and neurodegenerative marker deposition. Clinical phenotyping in our study considered the mere presentation of key manifestations, without accounting for variations in their severity. Gaig *et al.*^27^ recently proposed a composite score (ICS) for the clinical assessment of anti-IgLON5 disease that captures the extent and severity of various clinical manifestations. The ICS promises a superior quantification, allowing more intricate insights into the complex interplay of atrophy and clinical presentation. Finally, our study explored the possible effects of HLA-DQB1*05:∼ and immunotherapy on atrophy development, but data were only available from a limited number of subjects, and the results thus remain inconclusive. However, given the key influence of HLA-DQB1*05:∼ on genetic risk, age at disease onset and autoimmunity,^10^ future studies should further investigate possible differentiations in atrophy patterns subject to associated HLA haplotypes. Moreover, recent evidence indicates improved efficacy of immunotherapy when initiated early in the disease course^4^ and thus also a potential modulatory effect of immunotherapy on the progression of neurodegeneration and associated atrophy. However, given the retrospective and heterogeneous nature of the dataset presented in this study, including variability in treatment regimens, dosages, and timings relative to imaging, the ability to isolate and interpret immunotherapy-dependent effects is inherently limited.

Taken together, our study provides important contributions to the pathophysiological understanding, clinical monitoring and treatment of anti-IgLON5 disease. First, the findings extend the pathophysiological brain map of anti-IgLON5 disease by highlighting extensive and progressive atrophy at autoimmune and tau predilection sites. Remarkably, we identified very close spatial matching between causal pathophysiologic processes, including antibody and tau deposition, and brain atrophy. Second, these findings should stimulate further research into the molecular drivers of atrophy to derive effective therapeutic strategies to prevent its development and progression. Third, our results imply that atrophy could serve as a valuable marker for disease progression and in the clinical phenotyping of patients. Our results thereby call for the prompt incorporation of atrophy measurements into routine clinical assessments to monitor the disease trajectory and inform improved treatment strategies.

## Supplementary Material

awaf256_Supplementary_Data

## Data Availability

Raw data are available on request to the corresponding author.

## Appendix 1

### IgLON5 Imaging Consortium

Aurélie Méneret Isabelle Francillard, Dimitri Renard, Virginie Desestret, Nicolas Capet, Jeanbaptiste Davion, Julie Boucher, Helene Zephir, Philippe Damier, Helene Combres, Marie Rafiq, Fabrice Bonneville, Marina Cumin, Antoine Soulages, Mathilde Compoint, Maximilien Moulin, Edouard Berling, Eve Garrigues, Marine Chanson, Veronique Bourg, Caroline Giordana, Jeanne Benoit, Olivier Flabeau, Emmanuelle Derivoyre, Gilles Ryckewaert, Tifanie Alberto, Nicolas Carriere, Sohrab Mostoufizadehs, Melanie Barbay, Marie Benaiteau, Alberto Vogrig, Francois Sellal, Olivier Casez, Cedric Bruel, L Kalifa, Elena Camelia Rusu, Sarah Demortiere, Adil Maarouf, Adrien Wang, Adelaide Brasset, Clarisse Carra Dalliere, Arthur Attal, Céline Demourant, Florent Cluse, Jean-Luc Houeto, Norbert Brueggemann, Justina Dargvainiene, Thomas Seifert, Ulrich Hofstadt-van Oy, Michael Nagel, Christian Urbanek, Ina Schroeder, Peter Schramm, Silke Tonner, Caspar Seitz, Marlene Tschernatsch, Ha-Yeun Chung, Jonathan Wickel, Florian Schoeberl, Stefan Macher, Mateus M Simabukuro, Simone Zittel-Dirks, Brigitte Wildemann, Jan Kothaj, Carlos Ordas, Javier Villacieros Álvarez, Klemens Angstwurm, Stefan Macher, Evelyn Berger-Sieczkowski, Ángela Milán Tomás, Antonio Martin Bastida, Sonia Quintas, Nicola Tambasco, Pasquale Nigro, Raffaele Iorio, Yoya Ono, Takayoshi Shimohata, Kimura Akio, Takekoshi Akira, Mette Scheller-Nissen, Christian Hartmann, Danielle Bastiaansen, Robin van Steenhoven and Katia Schwichtenberg.
