# Supplementary material for: Brain atrophy patterns in anti-IgLON5 disease
Source: Brain. 2025 Jul 12;149(3):884–96. doi: 10.1093/brain/awaf256 (PMC13017494; doi:10.1093/brain/awaf256)
Supplement: awaf256_Supplementary_Data [file awaf256_supplementary_data.zip › brain-2024-02836-File009.pdf]

Brain Atrophy Patterns in Anti-IgLON5 Disease: Supplementary Materials

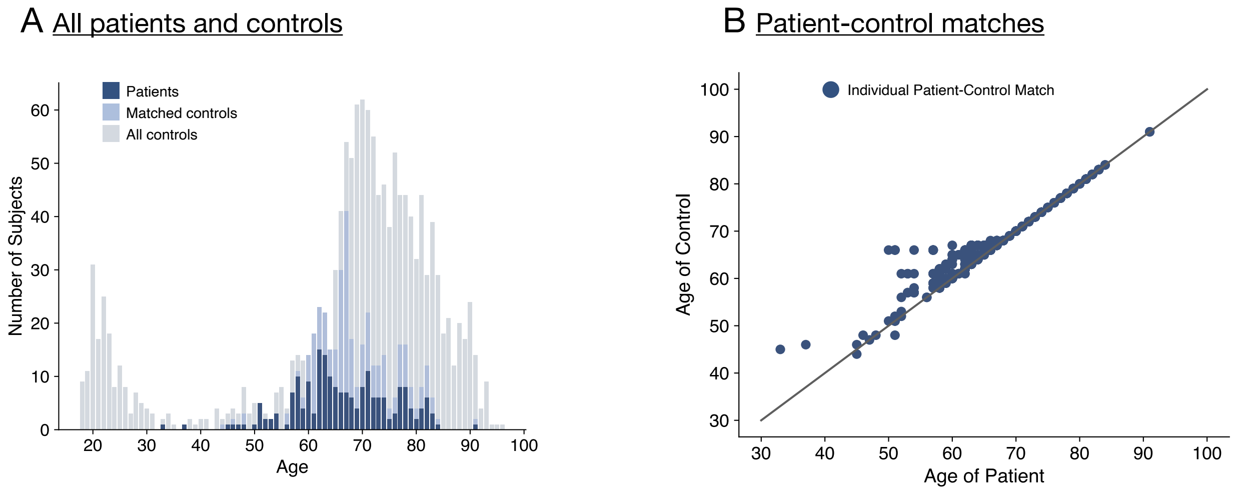

Supplementary Figure 1. Patient-Control Matching. (A) Overview of all controls (ADNI + OASIS repository) available for matching, matched controls and patients. **(B)** Matched patient-control pairs.

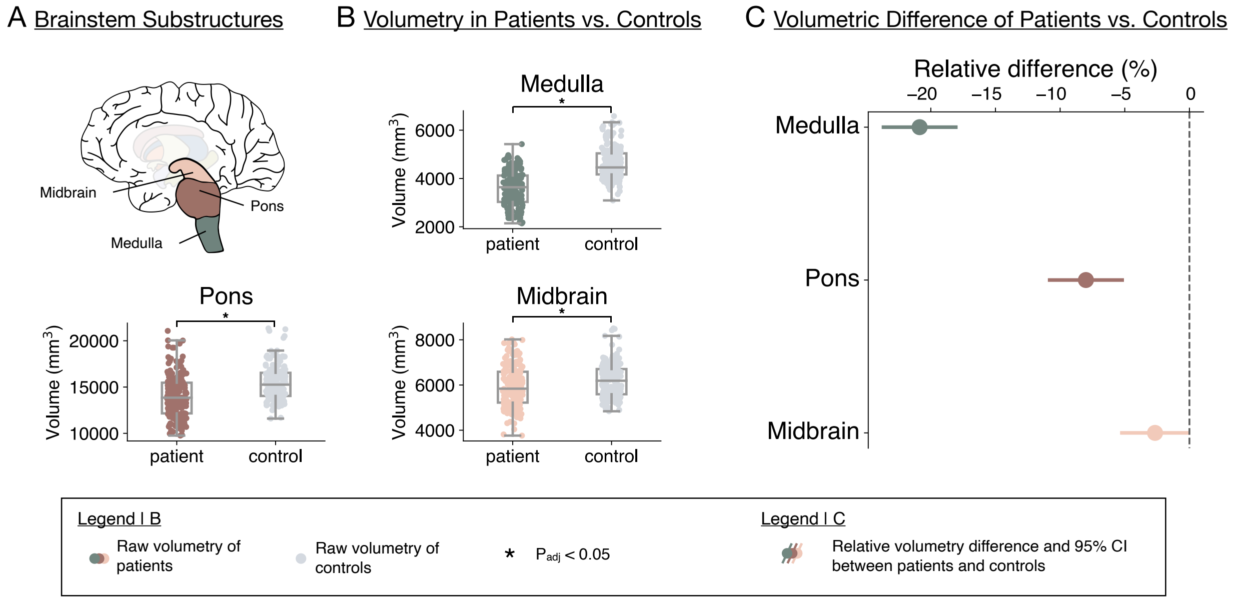

Supplementary Figure 2. Brainstem Substructure Volumetry of Anti-IgLON5 Patients. (A) Anatomy of brainstem substructures in their respective colour-coding that is carried forward in the remaining figure. **(B)** Region of interest (ROI) volumetry of patients (*coloured*) and matched controls (*grey*) shows significantly smaller volumetry in patients compared to controls in the

medulla, followed by pons and midbrain. **(C)** Relative difference and 95% CI (in %) in ROI volumetry between patients and matched controls shown in (B) *$* P_{adj} < 0.05$. For details on parameters included in analyses, see Supplementary Table 1.*

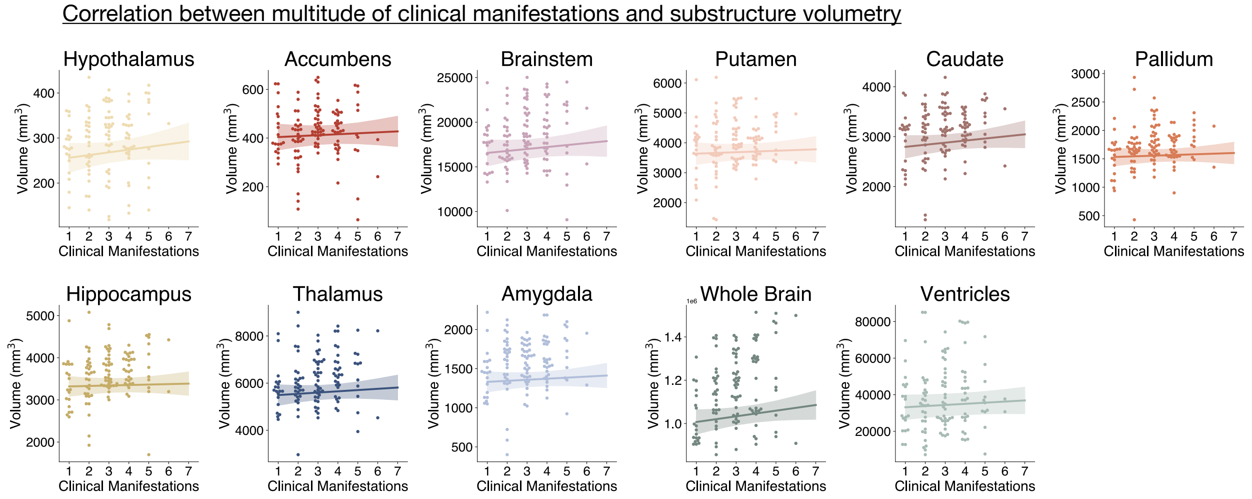

Supplementary Figure 3. Difference in volumetry stratified by multitude of clinical manifestations. Relationship of ROI volumetry in patients and multitude of clinical manifestations. Individual scatters show raw volumetry, lines the estimated relationship between volumetry and multitude of clinical manifestations and shaded regions the confidence interval. None of the relationships are significant. *For details on parameters included in analyses, see supplementary table 1.*

A Interaction of time since disease onset and clinical manifestation

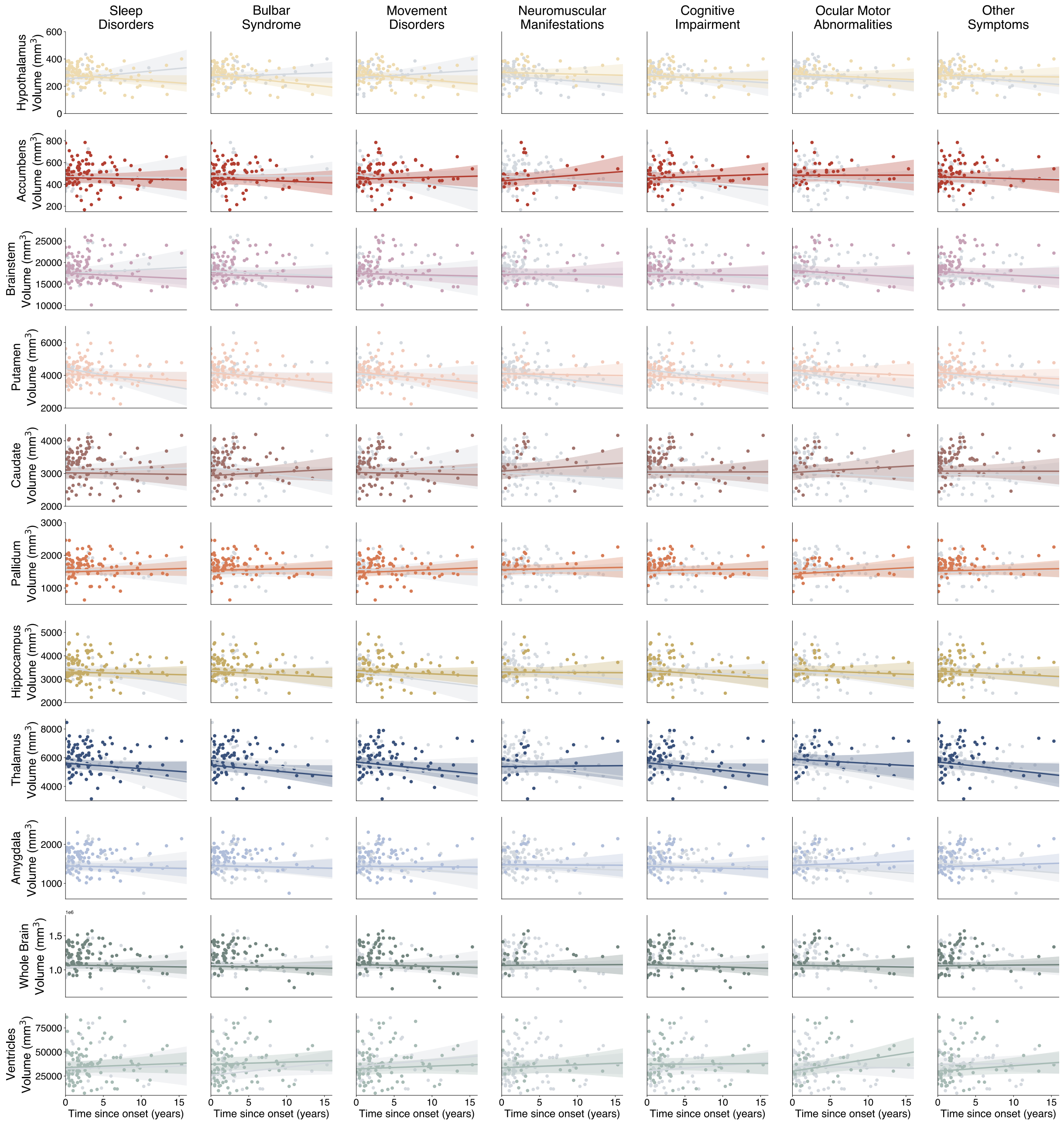

B Estimates and 95% CIs of interactions of time since disease onset and clinical manifestation

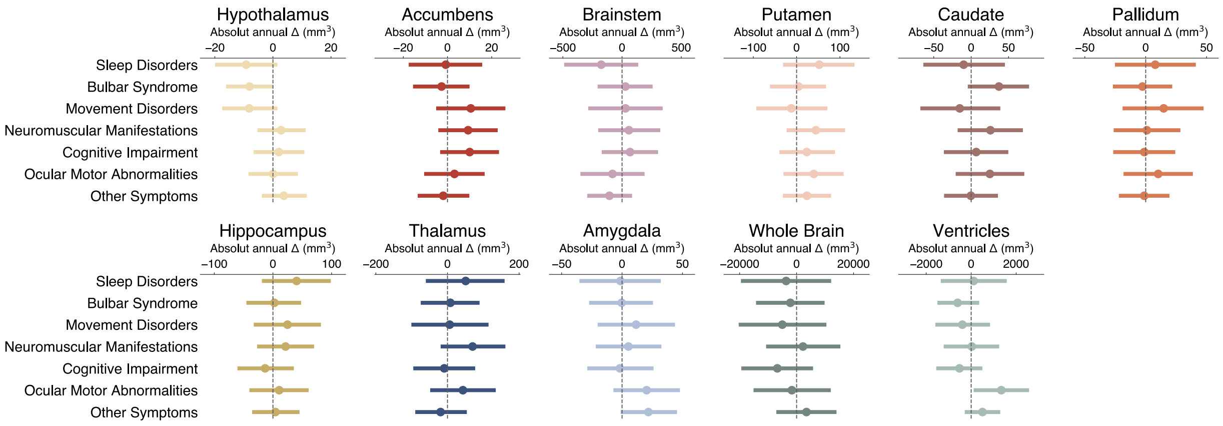

Supplementary Figure 4 [previous page]. Effect of clinical manifestations on longitudinal changes in volumetry. (A) Individual scatters show raw volumetry of patients presenting a specific manifestation (*coloured scatters*) or not (*grey scatters*). Lines show the estimated relationship between volumetry and time since disease onset, accounting for the interaction of time since disease onset and the specific clinical manifestation when presented (*coloured line*) and not presented (*grey line*). Shaded regions show the confidence intervals. **(B)** Estimates and 95% CIs of patients presenting relative to not presenting a specific clinical manifestation shown in A. *For details on parameters included in analyses, see supplementary table 1.*

Supplementary Figure 5 [following page]. Effect of *HLA-DQB1*05:~* on volumetry. (A) Volumetry of ROIs of non-carriers and carriers of *HLA-DQB1*05:~* is shown. **(B)** Absolute difference (mm^3) and 95% CI in ROI volumetry of carriers relative to non-carriers is shown in each paired plot on the right. **(C)** Individual scatters show raw volumetry of carriers (*coloured scatters*) and non-carriers (*grey scatters*) of *HLA-DQB1*05:~*. Lines show the estimated relationship between volumetry and time since disease onset, accounting for the interaction of time since disease onset and *HLA-DQB1*05:~* carrying (*coloured line*) and non-carrying (*grey line*). Shaded regions show the confidence intervals. **(D)** Estimates and 95% CIs of carriers relative to non-carriers shown in B. *For details on parameters included in analyses, see supplementary table 1.*

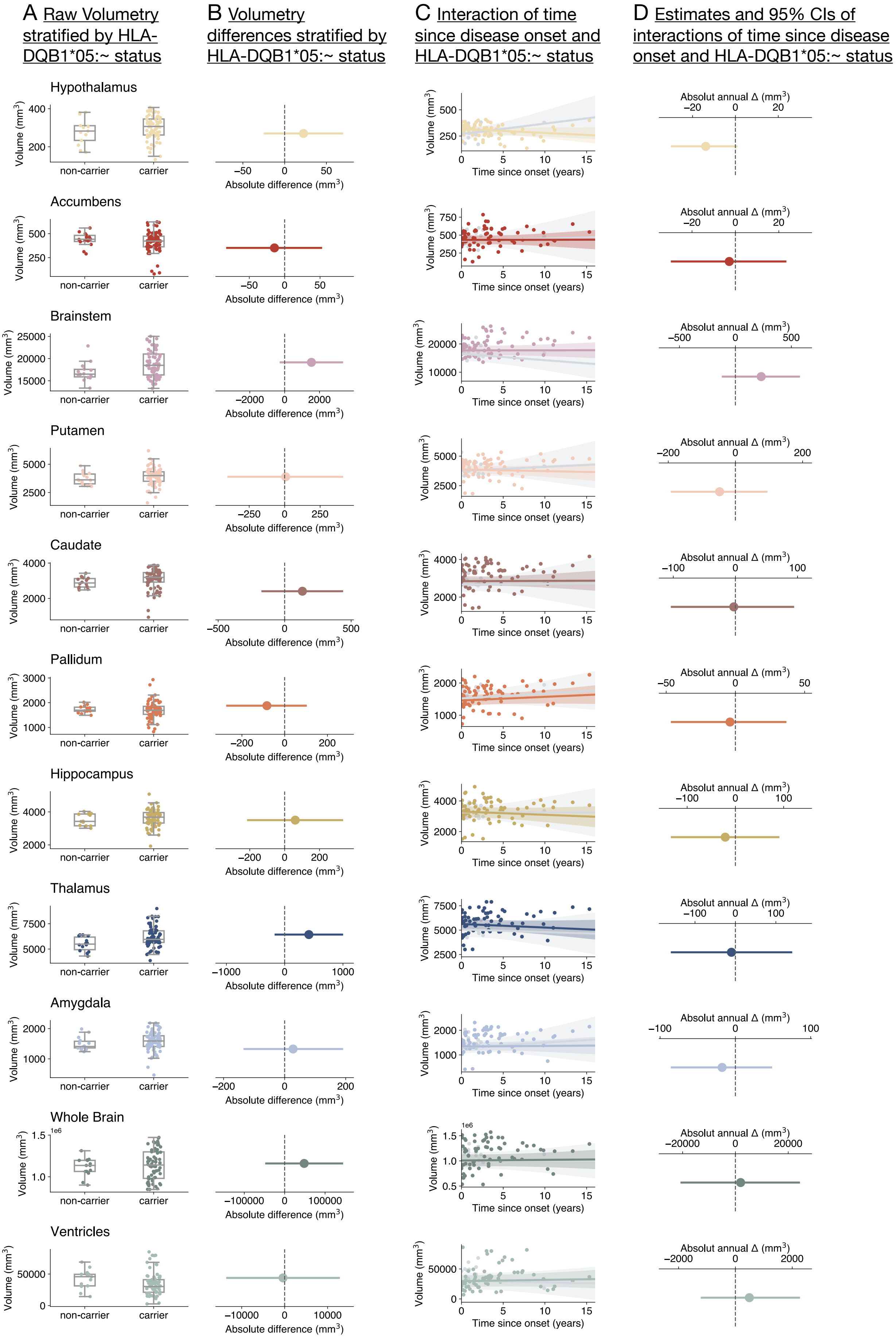
A Raw Volumetry stratified by HLA-DQB1*05:~ status
B Volumetry differences stratified by HLA-DQB1*05:~ status
C Interaction of time since disease onset and HLA-DQB1*05:~ status
D Estimates and 95% CIs of interactions of time since disease onset and HLA-DQB1*05:~ status
Hypothalamus
Accumbens
Brainstem
Putamen
Caudate
Pallidum
Hippocampus
Thalamus
Amygdala
Whole Brain
Ventricles
Volume (mm3)
non-carrier
carrier
Absolute difference (mm3)
Time since onset (years)
Absolut annual Δ (mm3)

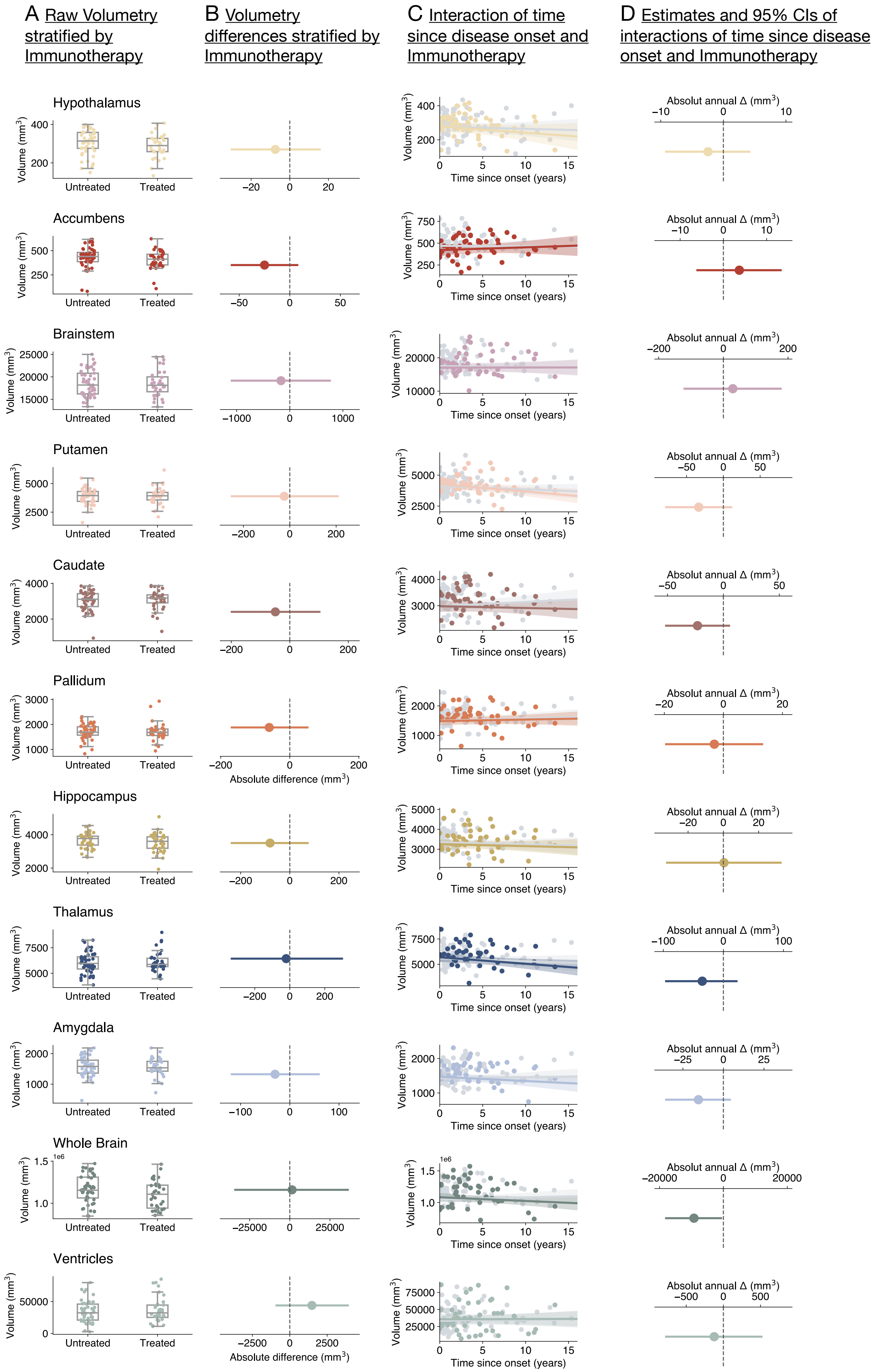
A Raw Volumetry stratified by Immunotherapy
B Volumetry differences stratified by Immunotherapy
C Interaction of time since disease onset and Immunotherapy
D Estimates and 95% CIs of interactions of time since disease onset and Immunotherapy
Hypothalamus
Accumbens
Brainstem
Putamen
Caudate
Pallidum
Hippocampus
Thalamus
Amygdala
Whole Brain
Ventricles
Volume (mm3)
Untreated
Treated
Absolute difference (mm3)
Time since onset (years)
Absolut annual Δ (mm3)

Supplementary Figure 6. Effect of immunotherapy on volumetry [previous page]. (A) Volumetry of ROIs of untreated and treated patients is shown. **(B)** Absolute difference (mm^3) and 95% CI in ROI volumetry of treated relative to untreated patients is shown in each paired plot on the right. **(C)** Individual scatters show raw volumetry of patients treated (*coloured scatters*) or not treated (*grey scatters*) with immunotherapy. Lines show the estimated relationship between volumetry and time since disease onset, accounting for the interaction of time since disease onset and immunotherapy when treated (*coloured line*) and untreated (*grey line*). Shaded regions show the confidence intervals. **(D)** Estimates and 95% CIs of treated relative to untreated patients shown in B. *For details on parameters included in analyses, see supplementary table 1.*
